# Situating Expertise: Lessons from the HIV/AIDS Epidemic

**DOI:** 10.1002/gch2.201700076

**Published:** 2018-06-19

**Authors:** Lauren Paremoer

**Affiliations:** ^1^ Department of Political Studies Rm 5.32 Robert Leslie Building University of Cape Town

**Keywords:** HIV/AIDS, scientific advisory committees, social determinants of health, social movements, South Africa

## Abstract

How do Scientific Advisory Committees (SACs) frame the relationship between political agency and expertise in their work? What are the political implications of the ways in which SACs legitimate or obscure specific forms of political agency? Using a South African case study, human immunodeficiency virus (HIV)/acquired immunodeficiency syndrome (AIDS) activists' participation in clinical trials designed to demonstrate the efficacy of highly active antiretroviral treatment (HAART) in resource‐poor settings, and the process of translating scientific knowledge about HIV/AIDS into public policy under the leadership of a SAC, the South African National AIDS Council (SANAC), is analyzed. The case study suggests that 1) political agency plays a significant role in generating and disseminating scientific data that allow activists to fulfill their political goals; 2) SACs primarily value political agency as a resource for implementing their prescriptions and legitimating their work; 3) processes of political conscientization, movement building, democratic collective action, and deliberation can contribute to the reliability and validity of the technical knowledge SACs rely on, and under some circumstances, contribute to the political resonance their recommendations have with impacted constituencies; and 4) social theory can serve as a resource for negotiating conflicts between technical experts and activists that cannot be settled by appealing to clinical facts.

## 1 Introduction

This paper explores the role of Scientific Advisory Committees (SACs) that involve nonscientists in decision‐making processes that use scientific evidence to formulate public health policies. I define SACs as a group of individuals with expertise on a specific issue that are mandated by decision makers in national governance institutions to give them nonbinding advice on a specific issue. The focus here is thus on SACs that enable citizen participation in governing science,^1^ but do not have the legal authority to give government institutions binding advice. Second, SACs as they are defined here, must give advice that is informed by evidence from the natural or social sciences, i.e., by contributory expertise.^2^ However, they may also draw on the interactional expertise, embodied knowledge, or metis of the nonscientists appointed to the SAC.^3^

I define policies broadly, i.e., as explicit and publicly accessible decisions and principles about how a public institution intends to define and address a specific issue. Policies, so defined, may not match what public institutions actually do. However, they do provide a useful gauge of government's attempts to articulate its visions of the relationship between science and politics that best serves the public interest. Here, I focus on public health policies informed by the advice of SACs, as a way of exploring two related questions. First, how do SACs frame the relationship between political agency and expertise in their work? Second, what are the political implications of the ways in which SACs decide to use or obscure specific forms of political agency?

Since the 1980s, citizen participation in governing science has expanded, particularly in North America and Europe.^4^ SACs represent a common institutional form for encouraging greater participation of nonexperts in scientific processes. Such participation has been defended on the grounds that it fosters greater trust and accountability in science and technology generally, and particularly in new knowledge emerging from these fields that may inspire anxieties among citizens about unintended consequences, unacceptable risks, or erosion of social norms when used as the basis for public policies.^5^ Others have criticized nonexperts' participation in governing science on the grounds that undermine technical expertise by eroding the distinction between scientific knowledge and popular opinion, or on the grounds that nonexpert participations in SAC processes are primarily used to legitimate or add nuance to the positions of politicians and/or technical experts.^6^ Underlying this disagreement is the more fundamental question of whether it is possible and desirable to disentangle science and politics. Collins and Evans have argued against the “tendency to dissolve the boundary between experts and the public so that there are no longer any grounds for limiting the indefinite extension of technical decision‐making rights” to a “technically qualified elite.”^7^ Though they acknowledge that this may increase the legitimacy of findings resulting from this process, they argue that this “problem of extension” is deeply problematic: it risks giving nonscientists, who may hold unreasonable and irrational but politically popular beliefs about technical questions, equal or greater priority in technical decision‐making processes than scientists.^7^ This may lead to irrational and ineffective public policies.

Epstein, on the other hand, has argued that attempts to separate science and politics amount to “misguided boundary work”.^8^ He argues that empirically grounded research in the field of Science and Technology Studies, as well as his own work on human immunodeficiency virus (HIV)/acquired immunodeficiency syndrome (AIDS) activism, demonstrates that in practice, science and politics are intertwined. For example, he argues that ACT‐UP activists' efficacy in influencing scientific research and public policy is “in part a product of [their] hybridity,” i.e., their ability to “routinely [combine] reasoned technical discourse with some of the most angry, outrageous, and sometimes casually reckless political speech and public theater found in any social movement of the late twentieth century”.^9^ Like Collins and Evans, Epstein argues against conflating science and politics. He makes the important point that activists are unlikely to be guilty of this when they themselves benefit from technical expertise that is sound enough to produce life‐saving technologies.

This paper seeks to contribute to this debate. It explores how political agency (defined below) might function as a resource for science and SACs, and how political agency is reshaped as a result of activists' engagements with SACs. It takes a constructivist approach in answering these questions. Building on the work of Braun and Schultz,^10^ I understand policymaking in the context of SACs, as well as the activism preceding this work, as processes through which participants construct novel forms of political agency. Like them, I am interested in “benefits, pitfalls, or unintended side effects” that emerge when activists embracing these forms of political agency are “enrolled” to do the work of SACs.^11^

## 2 The Case Study: HIV/AIDS Activism and SACs in South Africa

I explore the questions above with reference to a case study from the Global South: the participation of HIV/AIDS treatment activists who are members of the Treatment Action Campaign (TAC) in formulating successive National Strategic Plans (NSPs) for managing HIV/AIDS in South Africa (SA) by participation in a specific SAC, the South African National AIDS Council (SANAC). I revisit the literature on HIV/AIDS activism and policymaking in South Africa in order to identify the forms of political agency that were erased and affirmed through health activists' participation in SANAC, and the political implications of these dynamics. I choose to focus on the field of HIV/AIDS activism, and this particular case study, for a number of reasons. First, HIV/AIDS activism offers many examples of the impact that political agency has on public health and scientific practice. Significantly, these examples come from countries located in both the Global South and the Global North. Though the case may not offer any conclusive findings, it has the potential to generate further questions for comparative inquiry and about the specificities of SACs in the Global South, and I approach the analysis in this spirit. Below, I define the term “political agency,” and provide a brief overview of the literature that informs my decision to explore the relationships between political agency and SACs through this case study.

### 2.1 Political Agency and Health

It is now widely accepted that health is socially determined. This idea informs the work of several intergovernmental organizations working on health.^12^ The Whitehall Studies are regarded as some of the first to provide empirical evidence that a person's social status significantly contributes to his/her health status.^13^ The analysis presented here draws on this literature and assumes that political agency is one important social determinant of health. Following Brown, I define political agency as participation in institutions, organizations, or processes of “collaborative self‐governance.”^14^ Social movements that mobilize people to engage in “collaborative self‐governance” aimed at improving individual and public health is one site in which political agency is cultivated and expressed.

This conception of politics as collective action for the purposes of self‐governance echoes the conception of politics used by the Marmot Commission on the Social Determinants of Health (henceforth “the Marmot Commission”). It argues that the political agency of marginalized citizens can be magnified and consolidated through collective action, and that this political process is central to improving health outcomes, i.e.,

*“Theorizing the impact of social power on health suggests that the empowerment of vulnerable and disadvantaged social groups will be vital to reducing health inequities… Those concerned to reduce health inequities cannot accept a model of empowerment that stresses process and psychological aspects at the expense of political outcomes and downplays verifiable change in disadvantaged groups' ability to exercise control over processes that affect their wellbeing…Any serious effort to reduce health inequities will involve changing the distribution of power within society to the benefit of disadvantaged groups… This means that action on the social determinants of health inequities is a political process that engages both the agency of disadvantaged communities and the responsibility of the state*.^15^

Given this conception of power, one of the key overarching recommendations contained in the Marmot Commission's final report is to “reinvest in the value of collective action:” this intervention, rather than improvements in medical science or greater availability of health care services, is seen as foundational to addressing the inequalities in power, money, and resources that underpin health inequities.^16^ This paper explores whether SACs constitute a space in which disadvantaged groups can, in the Commission's words, effectively “exercise control over processes that affect their well‐being.”

The Commission is not alone in arguing that political agency shapes health outcomes. For example, research undertaken by Kark and Kark in South Africa in the 1940s showed that communities who take an active role in identifying and addressing their health needs through collective action and democratic decision‐making experience better health outcomes than communities who fail to do so.^17^ The Kark and Kark's work helped inform the Alma Ata Declaration's endorsement of the principle that “[t]he people have the right and duty to participate individually and collectively in the planning and implementation of their health care.”^18^ Unfortunately, this politically grounded conception of health failed to gain traction after the Alma Ata Conference, as the World Health Organization (WHO) adopted a more selective approach to primary health care.^19^

More recently, several studies have suggested that collective action by marginalized communities can translate into better health outcomes in these communities, and more reliable data about the health problems impacting them. For example, in her work on the Black Panther Party's health activism, Nelson argues that the Party's political work led to improvements in research on sickle cell anemia.^20^ Tuana argues that the US women's health movement, which emerged alongside the broader women's movement, can be understood as an “epistemological resistance movement geared at undermining the production of ignorance about women's health and women's bodies in order to critique and extricate women from oppressive systems often based on this ignorance,” while also improving their access to reproductive health services.^21^ With respect to HIV/AIDS science, political agency has proved particularly important in securing better health outcomes for people living with HIV/AIDS (PWAs). Epstein, reflecting on his research on HIV/AIDS activism in the US, has argued that the high levels of political mobilization among gay men prior to the outbreak of the epidemic contributed to their efficacy in securing investments in research on curing and treating HIV/AIDS.^22^ Biehl has written about HIV/AIDS stigma in Brazil as something that can cause “civic death,” a social status that ultimately contributes to the physical deaths of people living with HIV/AIDS – unless this stigma is countered by the presence of an “organized civil society” advocating for the well‐being of PWAs.^23^

Robins has argued that HIV/AIDS treatment activism contributed to the implementation of a public sector highly active antiretroviral treatment (HAART) programme in SA, thereby averting needless deaths. However, Robins emphasizes that this activism forged “a sense of collective solidarity and belonging” among PWAs that enabled them to give fuller expression to the citizenship rights they gained when SA became a constitutional democracy in 1994.^24^ Steinberg has argued that participation in ART support groups offered South African women a unique opportunity to forge distinctly feminized spaces for collective action, and that this was central to them participating in “the existential achievements of public participation. To speak and to act in a forum constituted by one's equals; to be seen to build and mend and fortify the foundations on which this forum stands… to imbibe an ancient and powerful experience of from which young women have since time immemorial been excluded.”^25^ These examples illustrate that political agency contributes to health outcomes by improving access to health care. However, this is but one component of a broader strategy aimed at affirming the status of activists and full and equal members of the political community who are entitled to participate in institutions of governance that affect their well‐being.

My argument is also informed by feminist critiques of science. Work in this field has convincingly demonstrated that our conceptions of scientific knowledge have historically perpetuated patriarchal, classist, and racist power relations, and that it has obscured or disavowed the contributions of lay experts and citizens in creating “expert” knowledge.^26^ This point has also been made by scholars in the field of Science and Technology Studies, for example, by Shapin and Latour.^27^ However, feminist scholars have made a distinctive contribution by not only exposing the political entanglements of scientific knowledge, but by arguing that this insight imposes an obligation on scientific or technical “experts” to adopt a praxis that dismantles the political and ethical biases that underpin their expertise. This focus on praxis is not necessarily emphasized by science and technology studies scholars. Feminist critiques of science thus make a notable contribution by making the political agency of scientists overt – and by encouraging them to be reflexive about this when engaging in knowledge production and dissemination.

Haraway's notion of “situated knowledges” is one prominent articulation of this position. In this paper, I build on her claim that “[r]ational knowledge is a power‐sensitive conversation” and it is important to expose the “confusion of voice and sight, rather than clear and distinct ideas” that informs knowledge production.^28^ How exactly can SACs be more transparent about the “confusions” that mark the policymaking process? One way of doing this would be to pay attention to the forms of political agency that inform scientific knowledge production and its applications to everyday life. At the very least, this involves acknowledging that processes of collaborative self‐governance that sustain or undermine knowledge production, and reflecting on how these forms of political agency can and should be incorporated into the work of SACs. It also requires that SACs be reflexive about the political implications of their interventions, i.e., that they consider how their recommendations bolster or undermine existing inequalities in power and resources that impede or enable citizens' ability to participate meaningfully in decisions about their well‐being.

## 3 A Clear Vision from Muddy Waters? Mapping SANAC's Efforts to Disentangle Political Agency and Expertise

This case study is organized around the work of two institutions, one a social movement and the other a SAC. The first institution is TAC, which is the most effective, enduring, and politically influential civil society organization advocating for HAART in SA. Its work has been informed by a sustained emphasis on training HIV/AIDS activists as lay experts endorse and are able to articulate the biomedical conception of HIV/AIDS and its management through antiretrovirals (ARVs).^29^ Between 1998 and 2008, i.e., the period of HIV/AIDS denialism, TAC was the government's chief antagonist because of its insistence that valid and reliable scientific data existed to prove that HIV caused AIDS, and that HAART is efficacious.^30^ During this period, TAC also served as a bridge between lay experts and technical experts (notably economists and biomedical researchers and practitioners) who were united in their desire to produce and defend scientific evidence of the affordability and clinical efficacy of HAART in resource‐poor settings.^31^ Below, I describe TAC members' roles in the scientific trials demonstrating the efficacy of HAART in resource‐poor settings, and discuss their framing of the relationship between political agency and technical expertise in the context of these trials.

The second institution I focus on is SANAC, which was established in 2000. SANAC is an advisory body responsible for formulating and overseeing the South African government's national HIV/AIDS strategy. Its key output is the National Strategic Plan for managing HIV/AIDS, tuberculosis (TB), and sexually transmitted infections (STIs), which serves as the overall framework guiding state and nonstate actors' interventions in these epidemics. Members of civil society, including HIV/AIDS treatment activists, are officially incorporated as SANAC members through their participation in its Civil Society Forum (CSF), which currently consists of 18 civil society sectors. In its current incarnation, five representatives of PWAs are included in the forum. The CSF is responsible for representing civil society in policymaking processes within the SANAC Plenary, which represents SANAC's political leadership and includes government ministers and is chaired by the Deputy President of South Africa, and its committees.

When it was first established, no medical practitioners, research scientists, or TAC members were included in SANAC.^32^ At this point, TAC was very critical of SANAC, given its reluctance at the time to oppose government's reluctance to implement programmes preventing mother‐to‐child transmission of HIV.^33^ SANAC was restructured in 2003 to include more civil society representatives. In the wake of this, TAC joined SANAC. TAC was especially important in revitalizing SANAC during a second round of restructuring in 2006, after which SANAC was co‐chaired by the then deputy president of TAC, Mark Heywood.^34^ As I illustrate below, many TAC activists are highly skilled “interactional experts” in matters of HIV/AIDS prevention and treatment. However, they also understand that their fluency in the biomedical language used to describe HIV/AIDS is one tool among many that can be used to forge the political agency of HIV/AIDS activists who are committed to improving treatment access and broader structural reforms that improve the social determinants of health. The case study below explores how the relationship between political agency and expertise is framed in SANAC's work, specifically in its most important output: the NSPs that serve as the keystone of South Africa's national strategy to curtail and control the HIV/AIDS epidemic. (In discussing the political economy of HIV/AIDS in South Africa, it is important to make a distinction between the pre‐2008 and post‐2008 period. In 2008, President Jacob Zuma entered office and appointed Dr. Aaron Motsaeledi as the Minister of Health. Both the officials were unambiguous in their support for a free public sector HAART programme. In contrast, the SA government's HIV/AIDS response prior to 2008 was shaped by the HIV/AIDS denialism of President Thabo Mbeki and his Minister of Health, Dr. Manto Tshabalala‐Msimang. Until about 2004, some in President Mbeki's government questioned the affordability and clinical efficacy of HAART. As a result, his government's efforts to ensure access to HAART in the public sector remained uneven and resulted in many deaths. The year 2008 marks a political turning point in the politics of the South African HIV/AIDS epidemic, as the affordability and clinical efficacy of HAART ceased to be politically contentious.) I argue that the case study illustrates that SANAC understood the significance and utility of political agency in much narrower terms than some of the activists represented in its structures.

### 3.1 Situating the Khayelitsha HAART Trials in the Global Political and Economy of Health

Between 1999 and 2003, Médecins sans frontières (MSF) and TAC demonstrated that a simplified and standardized HAART regime could effectively suppress viral replication in PWAs living in the South African township of Khayelitsha, which is characterized by high rates of household poverty, low rates of employment, and very poor infrastructure. The Khayelitsha HAART trials started in 2001 and enrolled patients who were diagnosed as being in Stage IV of HIV Disease or in Stage III with a CD4 count below 200 mm^−3^ into a standardized HAART regimen. They showed that HAART could radically improve the quality of life of PWAs, and that they remained treatment adherent, even in setting characterized by high levels of stress, relatively weak public health institutions (as compared to the standard of care available in the private sector), and high levels of physical and material insecurities. In the literature on HIV/AIDS treatment access, such settings became known as “resource‐poor settings.” The trial took place during a period of AIDS denialism in South Africa, and despite this, data from the trial eventually informed the Mbeki government's national treatment plan, which acknowledged the efficacy of HAART in treating HIV/AIDS. This was a significant national‐level victory.

The Khayelitsha trial was also pivotal in securing a global victory for treatment activists throughout the Global South. Prior to the trial, Big Pharma argued that the primary impediments to realizing universal treatment access in the “developing” world were weak public health systems and corrupt governments. In contrast, treatment activists argued that Big Pharma's insistence on extracting superprofits from the sale of ARVs was the primary impediment to universal access. Their ability to profiteer from ARV sales was enabled by the agreement on trade‐related aspects of intellectual property rights (TRIPS) Agreement of 1994, which introduced a new “world trading system, based upon open, market‐oriented policies.”^35^ TRIPS gives inventors of new processes or products patent protection for a period of 20 years.^36^ Though it gives national governments the authority to exempt “diagnostic, therapeutic, and surgical methods for the treatment of humans” from its provisions under conditions where these inventions are essential to protecting human life, governments in the South find it difficult to implement this exception.^37^

Treatment activists, particularly MSF, were immediately critical of TRIPS. In 1999, MSF therefore launched the Access Campaign to demonstrate that the newly established global patent protection regime, and its inflationary effects on the prices of ARVs, constituted a fundamental obstacle to accessing HAART in the developing world.^38^ At that time, pharmaceutical companies rejected the notion that patents and their inflationary effect on ARV prices were the primary obstacle to ensuring universal access to ARVs. Instead, they argued, the primary obstacles to treatment were poverty, failing public health systems, and corrupt and interventionist governments. Until these factors were corrected, states in the Global South would not be able to administer HAART effectively even if they could afford ARVs. In order to counter this argument, MSF decided to collect scientific evidence, demonstrating that weak public health systems and bad governance – what pharmaceutical companies referred to as “resource‐poor settings” – were not an impediment to effectively administering HAART. Khayelitsha was one of these settings in which it collected this scientific data to counteract the political rhetoric of Big Pharma. In the next section, I describe the trials that were conducted at Khayelitsha and their outcomes. In particular, I focus on the political organizations and social solidarities that led to the success of the trials.

### 3.2 Knowledge Making as an Expert/Nonexpert and Political Process: The Khayelitsha Trial

In April 2000, MSF in collaboration with TAC and Provincial Administration of the Western Cape (PAWC) set up three HIV/AIDS clinics in Khayelitsha's primary health care centers, providing prevention‐of‐mother‐to‐child transmission of HIV services (PMTCT services). In May 2001, these clinics began to offer HAART to people in the advanced stages of HIV infection.^39^ The MSF trials took place in Khayelitsha partly because it was regarded as a paradigmatic example of a resource‐poor setting and because TAC could provide psychosocial support for patients and political support for MSF and PAWC, who were operating in a hostile national political environment.^40^ This social movement was crucial to the sustainability of the trial, and the quality of data it generated. Participants had access to lay counselors and nurses who could help them formulate adherence plans or visit them at home. Crucially, participants in the trial were also given the opportunity to attend support grounds every fortnight that were only attended by people on HAART.^41^ This provided them a safe space in which to share the difficulties of adhering to their regimen and with living with HIV/AIDS. By 2003/2004, the Khayelitsha trials had conclusively proved that clinics could avoid therapeutic anarchy and achieve high levels of viral suppression in AIDS patients by following simple, standardized treatment regimens administered mainly by nurse practitioners. MSF later established a second HAART pilot site in Lusikisiki, a rural district in the Eastern Cape, in order to prove that HAART could be effective even in a rural area with almost no formal health care infrastructure. Here too, TAC activists provided vital HIV education and treatment support interventions.

Both TAC and MSF readily admit that lay health workers contributed immensely to the success of pilot programmes designed to demonstrate the feasibility of administering HAART in resource‐poor settings. Significantly, the treatment and testing support offered by a large cohort of volunteers, many of them TAC members, were integral to the successes obtained by the small number of doctors and nurses dispensing HAART through these pilot programmes.^42^ Volunteers took responsibility for a range of tasks aimed at assisting patients in remaining treatment compliant, including offering voluntary counseling prior to HIV testing, educating HIV‐positive patients about their disease and the science of HAART, and establishing treatment clubs or partnerships (i.e., becoming a “treatment buddy”) in order to support and monitor patients taking ARVs.^43^

TAC's volunteer programme was initially created with three objectives in mind, all of which would contribute to the success of the pilot programme. Volunteers would educate residents and medical practitioners at pilot sites about HIV/AIDS and HAART, would lobby for HAART services in the public sector, and would build a demand for HAART services by encouraging voluntary testing for HIV.^44^ By 2005, about 18 months into the implementation of the government's HAART programme, TAC's emphasis had expanded from building its own base of volunteers to additional efforts to secure paying jobs for its treatment literacy volunteers in the public sector programme:

*[TAC's] Treatment Literacy [programme] is hailed as a well run, timely programme aimed at creating the enabling environment for the rollout of an ARV programme in South Africa. Its key challenge is how to balance the need to compensate members for their work and at the same time retain the spirit of volunteerism. The Treatment Project provided a life‐line to members in need of treatment. With the rollout of the government's ARV programme there is a need for the Treatment Project to review its strategy and to exit members onto state programmes wherever possible.”* 45

TAC has been understandably proud of voluntary workers' sometimes referred to as “the nursing school of TAC,”^46^ contributions to the establishment of a HAART programme in a health system characterized by inadequate human resources and deteriorating physical infrastructure. In writing up the results of its Khayelitsha pilot programme, MSF recognized the contributions made by the civic‐minded TAC volunteers as establishing a new social contract between communities and service providers:

*“The community programmes of the TAC have educated many in the community about HIV/AIDS, prevention and ARV therapy (“treatment literacy”). In Khayelitsha, the link between education and treatment can best be described as an new social contract: the clinics provide effective HIV/AIDS care and life‐saving treatment, and the community breaks the silence, fights stigma and discrimination and, through education, promotes understanding and prevention.”* 47

Herman Reuter, an MSF doctor who helped to establish the Khayelitsha and Lusikisiki pilot programmes has argued that:

*“We [MSF] would not be here if it was not for TAC's mobilising and campaigning demanding a pilot site. Also we have 91–95% adherence on our ARV programme thanks to TAC.”* 48

Activists were important for securing the success of the pilot programme because they educated communities about HAART and created demand for it, they supported patients who were enrolled in the HAART programme, and because activists themselves made ideal patients because of their knowledge of HAART.^49^ It is worth emphasizing that volunteers undertook a wide range of tasks.

At the Lusikisiki site, for example, community caregivers ran HIV support groups and were responsible for ensuring treatment compliance by “directly observing” patients on HAART and by tracing and recalling defaulting patients. Two new categories of lay workers were introduced: “Adherence counselors” and “Support groups committees, activists, [and] people with HIV/ AIDS,” who prepared patients for treatment, ran support groups, educated the community about health promoting practices, collected data about HIV and HAART in the community, and advocated for better health care services.^50^ During the 2004/5 financial year, TAC's treatment literacy practitioners (TLPs) were also responsible for additional tasks including referral community members to health and social services providers, monitoring the availability of medicines at clinics, and participating in community structures, e.g., Clinic Committees, Health Forums, and District AIDS Councils.^51^ This use of lay health care workers was justified as being in step with health practices embraced by the most influential international organization responsible for public health, the WHO:

*“The World Health Organization promotes the role of primary health care and community‐led care in the delivery of antiretroviral therapy (ART) in resource [465] limited settings. In keeping with these principles, the delivery of HIV services in Lusikisiki was achieved through decentralization to primary health care, task shifting within services, and strong community support… With appropriate training, mentoring, and supervision, it was possible to delegate the running of the ART program to primary health care nurses and community health workers [in Lusikisiki]”* 52

The broader political context in which the Khayelitsha treatment trial played out was equally significant as it gave HIV‐positive persons, and specifically trial participants, a sense that they were contributing to a bigger struggle to create a more just postapartheid order. Contributing to knowledge making about HAART was this understood both as a means to improve their own vitality, and that of a more equal and just society. TAC's strategies for realizing the right to treatment have included mass mobilization, popular education, litigation, and volunteerism. Its work has been driven by the desire to demonstrate the competencies of citizens – regardless of their income level or formal educational experiences – to understand the complexities of living with HIV and of adhering to HAART regimes, and to affirm their rights to participate in decisions about public affairs, particularly with respect to HIV/AIDS treatment and care.^53^

As already mentioned, one of the organization's most important campaigns is its treatment literacy programme which it describes as giving “ordinary people – many of them with little formal education – an in‐depth understanding of the science, politics, and treatment options for HIV/AIDS” and as ultimately giving them an “understanding of health, HIV, and HIV medicine, …the politics of treatment access, and the essentials of activism.”.^54^ After the announcement of the public sector HAART programme TAC “concentrated on building the necessary human, community, and other resources to stimulate activism for ARV delivery in local communities,” thereby enabling trainees to “serve as community resources, helping to create a demand for treatment at each site.”^54^

The organization thus saw its political training and organizing work, i.e., building informed and active citizens, as the foundation for creating reliable knowledge about the conditions under which HAART can succeed in resource‐poor settings. These conditions involved more than simply biomedical interventions – it also hinged on living in communities that fought HIV stigma, and on having the opportunity to join an organization such as TAC that gave poor and marginalized citizens the opportunity to produce knowledge about HIV that was not divorced from their everyday challenges and experiences. In any comprehensive reading of the findings from the Khayelitsha trial (and also the Lusikisiki trial introduced later), it is clear that treatment success depended on the social solidarities and political organization established through TAC, and not only on the efficacy of a particular biomedical intervention designed by scientific experts. Stated differently, a comprehensive reading of “evidence” from the trial data suggest that lessons about clinical case management and about movement building and political organization would have to inform evidence‐based policies on how to replicate the trial's successes in other resource‐poor settings.

In the next section, I discuss how SANAC, a SAC that TAC activists have participated in since its inception, has framed the significance of political agency in the four NSPs it has produced to date. I argue that SANAC has framed the political agency of activists as an important resource for legitimating its own work, and for supporting the implementation of the government's NSPs. However, it obscures the importance of political agency in forging activists' confidence in their ability to determine the forms “collaborative self‐governance” they wish to prioritize in order to achieve improvements in the social determinants of health.

### 3.3 Repurposing Political Agency for Policy Implementation: Conceptions of Political Agency in the NSPs

SANAC has published four NSPs since its inception in 2002, all of which have been formulated in consultation with treatment activists who are also SANAC members.^55^ In the discussion below, I refer to these plans as NSP 1, NSP 2, NSP 3, and NSP 4. All the four plans emphasize the importance of individual responsibility, often articulated as “behavioral change,” in contributing to HIV prevention and HAART adherence. Significantly, only the third report strongly emphasizes the importance of curtailing the epidemic by improving the social determinants of health of most South Africans,^56^ and explicitly acknowledge that political determinants of health contribute to increased “vulnerability” to contracting HIV, though it does not specify how they do so.^57^ NSP 4 speaks about “structural drivers,” “structural factors,” and “social and structural determinants” of vulnerability.^58^ Toward the end of the document, “political factors” are identified as one of the factors that “make a person more vulnerable to HIV/AIDS.”^59^ However, structural factors such as socioeconomic status, migration, gender, and substance abuse are more consistently mentioned throughout the document as the key “social and structural determinants” of health.^60^

Collective action, citizenship, mass mobilization, social movement, and power are terms rarely used in these policy documents. However, the NSPs frequently include references to the need for political leadership as an essential component of the government's HIV/AIDS response, and to empowering PWAs, “communities,” and “civil society” to adopt health‐promoting behaviors. The latter two terms are not clearly defined in any of the NSPs, nor connected to a theory of change, explaining the link between community empowerment and improvements in the social determinants of health.

More typically, all the four NSPs frame “communities” as one of the most efficient sites for delivering HAART services. These efficiencies derive from the fact that communities are presumed to contain pre‐existing support mechanisms that augment the interventions laid out in the various NSPs. For example, the first NSP speaks about the importance of “community‐based support programmes” that government can utilize in order to improve treatment adherence rates.^61^ Communities are thus framed as sites where psychosocial support to PWAs can be obtained for free or low cost, i.e., on a voluntary basis. NSP 4 also acknowledges the importance of peer support in promoting adherence, but this is framed as something that can be cultivated through government “investment,” with no explanation of how these investments relate to already existing activist organizations working in this field, i.e.,

*“Under this NSP, South Africa will invest greater resources and effort in the training and mobilisation of peer educators, lay counselors and support personnel. Peers have an especially vital role to play in contributing to the response for young people and for other key and vulnerable populations. To play their optimal role, peer workers require effective training, support and supervision, and stipends or other compensation.”* 62

Neither this NSP nor any of the others acknowledge the political mobilization by TAC or other health activist organizations that established these psychosocial support mechanisms, the funding shortfalls that impede their ongoing work in this area,^63^ or the potential for political disagreement between government and health activists about how and why peer support – something TAC describes as a mechanism for “building local activism” – is politically important.^64^

The NSPs also define the community level care as a potential site of cost savings, given that it is more affordable to provide HAART services “at the community health center level” rather than in hospitals.^65^ NSP 2, for example, acknowledges that “[c]ommunity and home‐based care have grown rapidly in South Africa in the last five years.”^66^ As a result, the government developed mechanisms to institutionalize this form of care, e.g., introducing guidelines, training, and stipends for home‐based caregivers.^66^ Aside from emphasizing that community‐based care lowers the costs of rolling out HAART, NSP 2 argues that the government's stipend for caregivers “also contributes to poverty alleviation” and to individual upward mobility through creating entry‐level jobs in the public health sector.^66^

This reliance on communities as poorly paid or unpaid providers of basic health services has only intensified over time.^67^ NSP 4, the most recent plan, doubles down on the government's commitment to utilize community health workers (CHWs). It recommends that “[c]ommunity health workers should be formalized as a cadre, appropriately trained and supported, and fully integrated into the health system.”^62^ None of the NSPs acknowledge that stipends for home‐based carers are paid erratically, that these stipends are minimal,^68^ and that caregivers are not provided with any materials to assist them in their work. Most significantly, none of the NSPs acknowledge that many home‐ and community‐based caregivers working with PWAs during the early days of the epidemic were politicized through this work, i.e., that it has been central to building their political agency, and that much of their interactional expertise regarding HIV/AIDS was acquired through their political work in TAC.^69^

Inequalities of power and social status are acknowledged in the NSPs as important constraints on SANAC's work. These inequalities cause tensions within the organization. Among civil society representatives, those that possess interactional expertise, have more economic resources, greater knowledge of how government institutions and funding bureaucracies work, bigger membership bases, and working relationships with technical experts outside of SANAC structures seem to exercise more influence over SACs ongoing organizational work and outputs – and to be more vocal about its shortcomings during periods when senior politicians sideline SANAC.^70^ This has sometimes led to conflict and resentment among its civil society representatives, which has complicated their working relationship within SANAC.^71^ This sentiment has been particularly acute during period when SANAC has been dysfunctional due to conflicts amongst senior politicians, or during periods where civil society organizations compete for a declining pot of funding from donors.

Only one NSP explicitly mentions the enervating effect these tensions have on SANAC's civil society partners. In NSP 4 the “Message from the SANAC Vice‐Chairperson” states that the Council should “reject the ideology of contributing to shrinking the civil society space” and should recognize “that SANAC is an association where many members are volunteers and must be recognized for their expertise, time, and contribution.”^72^ However, the main text of the plan does the opposite: it notes that “[a]n agile, well‐resourced civil society is better positioned to contribute to stronger community systems and to ensure a seamless continuum of care from the health to the community system”^73^ but sets out no strategy for achieving this kind of civil society.

The four NSPs' repeated focus on building the capacity of civil society arguably amounts to an implicit acknowledgement that SANAC operates in a national context defined by an overburdened and organizationally weak civil society sector. This undermines the efficiency and efficacy of involving “civil society sectors” in its structures, and limits its ability to rely on them in realizing the NSPs. Though the capacity building initiatives contained in the NSPs may address these constraints, they are also likely to encourage forms of collective action that bolster the efficiency and legitimacy of SANAC. For example, capacity building is mainly taking place in the civil society “sectors” that correspond to SANAC's own organizational structures (e.g., the sectors represented on it), and for the purpose of building and maintaining political support for the NSPs' priorities and commitments – even when these are in tension with the priorities and commitments of some of SANAC's members.

Criticisms of the most recent NSP provide a clear illustration of this. The outgoing chief executive officer (CEO) of SANAC, Dr. Fareed Abdullah, has characterized the context in which the most recent NSP was written as one where dissenting opinions are discouraged and “sycophancy… overpowers the work of government.” As a result, he argues, the most recent NSP is the result of a process that allowed for “cherry picking [of] technical work with the aim of making government look good while at the same time giving just enough airtime to the latest evidence to avoid criticism from technical constituencies inside and outside the country.” Abdullah criticizes the plan for allowing “South African super‐non‐governmental organisations (NGOs), also known as the President's Emergency Plan for AIDS Relief (PEPFAR) partners… to scale up test and treat in public sector facilities in 27 high‐burden district municipalities in the country… [while] not even offer[ing] a minimum package for persons outside the 27 high burden districts for any HIV prevention or treatment services.”^74^ This uneven implementation of HIV/AIDS services violates the right to equal treatment enshrined in the South African constitution. It also distorts the civil society landscape by directing financing toward civil society organizations who are willing to act as a “service delivery” arm of government and its donors, even when these donors – like PEPFAR – prohibit local NGOs represented on SANAC structures from implementing key aspects of the NSP (e.g., advocating for the nondiscrimination and decriminalization of key populations like sex workers). On the basis of these and other shortcomings in the fourth NSP, and concerns about corruption within SANAC, TAC has warned that it might stop participating in this structure.^75^

Significantly, none of the NSPs acknowledge the importance of political organizations or social movements in serving as training grounds for cultivating the interactional expertise that facilitate activists' access to SACs, or as co‐producers of the scientific data that SACs rely on to do their work. Instead, the NSPs frame PWAs and “key populations” in passive terms, as groups to be studied, and not as active collaborators in producing new knowledge about HIV/AIDS. This absence is striking as collective actions by TAC activists, many of whom participate in SANAC as “civil society representatives,” were crucial to producing data, demonstrating the feasibility and affordability of treatment in SA. Despite this, the NSPs repeatedly describe “research” as a process that as the apolitical domain of technical experts and the research field as something that is pre‐existing and not, as in the Khayelitsha case study suggests, something that has to be created through collective action. The political dimension of research is acknowledged only in the sense that the NSPs warn against research agendas becoming too donor‐driven,^76^ and too disconnected from the needs of communities.^77^

## 4 Conclusion

What does TAC's involvement in the Khayelitsha trials and SANAC's subsequent efforts to harness this data highlight about the benefits, pitfalls, and unintended side effects of health activists' participation in SACs? A key insight from the case study is that TAC member's sense of political agency was central to their ability to successfully collaborate in the knowledge production processes that occurred in Khayelitsha and later in Lusikisiki. This sense of agency was not only derived from belonging to a mass movement, i.e., the ability to translate organizational numbers into demands for representation or inclusion in clinical trials. It was also grounded in TAC activists' interactional expertise, i.e., their ability to collaborate effectively and authoritatively in the research process. With very little material resources, TAC activists successfully pioneered adherence support, and assisted in recruiting trial participants. Perhaps most significantly, they used their collective power to create the political space for experimental clinical trials to be conducted in a poor community, and for this to be seen as a legitimate practice and a public “good.” This is a remarkable achievement, given the suspicion toward HIV/AIDS science at the time, and the misgivings marginalized communities have historically expressed about their knowing and unknowing participation in experimental medicine and clinical trials.^78^ Equally important is the fact that activists' interactional expertise was acquired through a political process aimed at informing and politicizing them. TAC's successful collaboration in the Khayelitha trials shows that these pedagogies succeeded in giving activists interactional expertise, but also reframed a deeply stigmatizing and private experience – living with HIV/AIDS and/or in an HIV/AIDS‐affected community – into a basis for acting in solidarity with similarly affected people, and with other marginalized groups (e.g., people living with TB, patients in the dysfunctional public health system, sex workers, and victims of xenophobic violence).^79^

A review of the NSPs suggests that SANAC recognizes the organizational reach and political agency of treatment activists, but that it tends to understand them in fairly narrow terms, i.e., as resources for implementing its own plans. It has not shown any clear commitment to incorporate movement building or mass mobilization for the right to health into its framework for addressing the high burden of HIV/AIDS, TB, and STIs that the NSPs seek to prevent and manage. The case study suggests that the political agency of its activist members can help to legitimate the institution, support its daily functioning, and subsidize the implementation of its premier output, the NSP. The case suggests that these legitimation and efficiency effects are likely to be most effective during periods when SANAC members share a consensus about a specific course of action, and in the absence of perceptions that some members – be they politicians, technical experts, or activists – have arbitrary or disproportionate power in shaping the advice SANAC shares with government.

However, SANAC's position on the negative potentialities of involving activists in its processes is less clear. There is empirical evidence to suggest that activists' involvement in SANAC may come at the expense of their ability to invest their time and expertise in their own organizational processes. Capacity building interventions or increased funding for weaker SANAC members does not necessarily address this conundrum, as this may result in their financial dependence on SANAC or external institutions that facilitate access to such resources (e.g., donors or government capacity‐building initiatives). This may well place pressure on civil society organizations to align with the political and ideological agendas of benefactors, and undermine their ability to take political direction from their membership base.

How might the pitfalls of engaging activist organizations in SACs be avoided? What can SACs do to remain alert to their power to distort and undermine forms of collective action that sometimes assist them in their work, and at other times threaten their legitimacy and efficacy? The case study suggests that the distinction between making facts and using or applying facts is an important one to keep in mind when studying the interplay of politics and science in the context of SACs.

Political contestations about making facts center on which facts can and should be produced through scientific research. The case of the Khayelitsha trials suggests that once such decisions are settled and translated into a legitimate research process, both experts and nonexperts seem equally invested in defending the “incontestability” of the scientific findings. There is a shared trust in both the research process and its findings. This is a tentative conclusion, especially because the Khayelitsha trials produced the outcome that activists and technical experts were looking for. Of course, this dynamic may hold even when common questions and legitimate processes produce research findings disappointing both researchers and activists: for both activists and technical experts, working with “trusted” facts increases their ability to gain an audience with and make demands on institutions that derive their authority from “evidence‐based decision‐making,” e.g., courts, state agencies, intergovernmental organizations, and possibly donors.

Political contestations about which facts to “make,” i.e., which scientific questions societies should prioritize, offer an example of using political agency as a creative force in science, rather than a distorting or disrupting force. In the Global South, where research agendas are often donor‐driven, SACs – especially domestically funded SACs – could potentially offer an institutional home for amplifying this creative political work of publicly and legitimately clashing about the scientific questions and facts that matter most in specific societies. This is one modest way of responding to Haraway's injunction that we expose the “confusion of voice and sight, rather than clear and distinct ideas” that informs knowledge production, while at the same time situating or grounding these confusions in the political communities that give rise to them.

The second type of conflict centers on how facts should be used – or whether they should be used at all. Such conflicts may center on trusted facts, or facts that are regarded as dubious. These conflicts cannot simply be settled by appeals to technical knowledge, as they involve normative questions. The case study suggests that in the Global South, these conflicts are only heightened by the fact that external actors like donors play a greater role than in the North in shaping government and civil society perceptions of which facts are “relevant,” and how they should be incorporated into policies. SACs like SANAC have not been immune to these pressures, and it strains the legitimacy of the institution and the advice it proffers.

If SACs cannot appeal to clinical or biomedical facts to settle these kinds of conflicts, what other principles or forms of knowledge can they appeal to? I would argue that SACs should draw on social theory, particularly critical social theory, and the forms of evidence and analysis it embraces in negotiating these conflicts. The Marmot Commission Report, with its emphasis on the health effects of power and collective action, arguably represents one example of how SACs can do this kind of work. Its findings suggest that SACs mandated with promoting public health should not prioritize clinical “facts” at the expense of ignoring or eroding the political agency of the most marginalized members of society. In doing so, they risk undermining their own long‐term goals. More broadly, the case study suggests that processes of political conscientization, movement building, democratic collective action, and deliberation at the local, national, and global scales can contribute to the reliability and validity of the technical knowledge SACs rely on, and under some circumstances, to the political resonance their recommendations have with the constituencies impacted by this knowledge.

## Conflict of Interest

The authors declare no conflict of interest.
